# Editorial: Updates on immunity to influenza A virus in humans and animals

**DOI:** 10.3389/fimmu.2022.1115406

**Published:** 2022-12-30

**Authors:** Irene Ramos, Aitor Nogales

**Affiliations:** ^1^ Department of Neurology, Icahn School of Medicine at Mount Sinai, New York, NY, United States; ^2^ Precision Immunology Institute, Icahn School of Medicine at Mount Sinai, New York, NY, United States; ^3^ Animal Health Research Centre (CISA), Centro Nacional Instituto de Investigación y Tecnología Agraria y Alimentaria (INIA, CSIC), Madrid, Spain

**Keywords:** influenza A virus, innate immunity, antiviral, adaptive immunity, inflammation

In this Research Topic, we compiled four original research articles where authors investigated different aspects of the innate or adaptive immune response to influenza A virus (IAV) infections. A balanced innate immune response is necessary to promote virus clearance and the development of adaptive immune responses. On the other hand, an exaggerated immune response causes tissue damage and a deficient immune response is unable to control virus spread. For this reason, understanding the mechanisms regulating all the different aspects of the immune responses is critical. IAV has developed multiple and sophisticated strategies to counteract the defense mechanisms triggered by the host innate immune system, including the production of interferon (IFN) and the activities of IFN-induced host proteins that inhibit virus replication. Multiple cell types play important roles during IAV infection, dissemination, and induction of host responses to control the virus. In addition, IAV strains differ in their ability to counteract host responses. These differences among cell types and IAV strains need to be systematically investigated.

Zhou et al. studied the mRNA expression pattern and molecular responses among distinct human cell types infected with various subtypes of IAV, using data from the gene expression omnibus (GEO) repository at the National Centre for Biotechnology Information (NCBI). Authors observed globally similar patterns of induction of gene expression among different cell types upon infection. Host factors such as IFIT2, ISG15, HERC5, RSAD2, GBP1, IFIT3, IFITM1, LAMP3, USP18, and CXCL10 could be key proteins in alveolar basal epithelial cells against IAV infection, while BATF2, CXCL10, IFI44L, IL6, and OAS2 seem to play more important roles in airway epithelial cells. Moreover, some overlap in upregulated genes between human primary epithelial cells infected with high or low pathogenicity IAV strains were shown. This kind of studies will help to better define the patterns of molecular responses induced during IAV infection and identify targets for novel antivirals.

Signal transducer and activator of transcription 2 (STAT2) is an important transcription factor that plays an essential role in immune responses to viral infection (1). Moreover, it has been described that disruption of STAT2 expression impairs the innate antiviral response, leading to higher viral replication levels (2). Li et al. found that early phosphorylation of STAT2 at position Y690 can be induced by different IAV strains and dsRNA or DNA viruses. Importantly, this activation of STAT2 was independent of type I IFNs and JAK kinases, but it was regulated by the RIG-I/MAVS pathway. In addition, authors show that upon IAV infection, Syk and MAPK12 (mitogen activated protein kinase 12) may be involved in the early activation of STAT2 through Y690 phosphorylation. Inhibition of MAPK12 kinase decreased the expression of multiple interferon-stimulated genes (ISGs), thus facilitating IAV replication. These findings suggest a new molecular mechanism by which early activation of STAT2 mediates antiviral responses to block IAV infection.

The study by Dorna et al. focused on the effect of the receptor binding specificity of IAV in the activation of human immune cells. Zoonotic IAVs are of great concern due to occasional outbreaks in domestic animals, poultry and in humans, as well as their pandemic potential (3, 4). Human and avian IAV differ on the type of sialic acid (SA) receptors their hemagglutinin (HA) interacts with. While human IAVs preferentially bind α-2,6 linked SA, avian IAVs preferentially bind α-2,3 linked SA (5). This differential receptor use, together with HA stability, are important determinants of the IAV tropism (5, 6). A previous study showed different activation of dendritic cells (DCs) by IAVs with different receptor specificity (7). To better understand the role of the IAV HA type in the activation of human immune cells, the authors analyzed the ability of several well characterized IAVs to infect and activate cells isolated from human peripheral blood mononuclear cells (PBMC). Monocytes and lymphocytes were in general less susceptible to infection by IAVs with avian-like (α-2,3) than human-like receptor (α-2,6) binding, while DCs showed similar levels of susceptibility. Interestingly, surface levels of α-2,3 SA or α-2,6 SA mapped to the infectivity by IAV with avian-like and human-like HA binding, respectively. IAVs with avian-like (α-2,3) binding HA showed stronger activation of PBMCs with enhanced cytokine expression. Furthermore, a protective role for high basal levels of IFITM3 expression in monocytes against IAVs with conformationally stable HA was identified. This study provides evidence that the HA type of IAV has an impact on virus’ ability to infect and activate human innate immune cells.

Hao et al., investigated the role of the transcription factor Runx3 in immunity to IAV. Interestingly, they found that while abrogating the expression of Runx3 *in vivo* had limited effects on virus clearance and survival, it had significant effects in the modulation of the immune response. A role for Runx3 in the development of lung antigen-specific CD8+ cells was identified. On the other hand, mice lacking Runx3 expression showed increased numbers of myeloid cells in the lung, and higher levels of pro-inflammatory cytokines in the bronchoalveolar lavage fluid (BALF) as compared to control mice. Strong upregulation of Runx3 was found in CCR2 expressing cells (monocytes and macrophages) in the lungs from infected mice. In BALF cells, expression of Runx3 was increased after IAV infection. Overall, results from this study support an important role for Runx3 in the regulation of the immune response to IAV with, intriguingly, very limited effects in protection. One explanation, as speculated by the authors, is that there might be compensating effects from different arms of the immune system, i.e. changes in the numbers of lung myeloid cells might compensate for loss of antigen-specific CD8+ T cells. An open question is how the loss of CD8+ T cells by Runx3 depletion would affect protection against a secondary IAV infection, which might provide evidence for a role for Runx3 in protection through adaptive immunity.

## Concluding remarks

The manuscripts in this Research Topic present exciting findings and insights to expand our understanding of the molecular basis of immune responses induced upon IAV infection. This information helps us clarify key aspects related with viral-host interactions and the mechanism of action of host effector molecules and cells involved in defense machineries.

## Author contributions

All authors made a substantial, direct, and intellectual contribution to the work and approved it for publication.
